# miR-132 Enhances Dendritic Morphogenesis, Spine Density, Synaptic Integration, and Survival of Newborn Olfactory Bulb Neurons

**DOI:** 10.1371/journal.pone.0038174

**Published:** 2012-05-31

**Authors:** Manavendra Pathania, Juan Torres-Reveron, Lily Yan, Tomoki Kimura, Tiffany V. Lin, Valerie Gordon, Zhao-Qian Teng, Xinyu Zhao, Tudor A. Fulga, David Van Vactor, Angélique Bordey

**Affiliations:** 1 Departments of Neurosurgery, and Cellular and Molecular Physiology, Yale University School of Medicine, New Haven, Connecticut, United States of America; 2 Department of Neuroscience, University of New Mexico School of Medicine, Albuquerque, New Mexico, United States of America; 3 Department of Cell Biology, Harvard Medical School, Boston, Massachusetts, United States of America; University of Michigan, United States of America

## Abstract

An array of signals regulating the early stages of postnatal subventricular zone (SVZ) neurogenesis has been identified, but much less is known regarding the molecules controlling late stages. Here, we investigated the function of the activity-dependent and morphogenic microRNA miR-132 on the synaptic integration and survival of olfactory bulb (OB) neurons born in the neonatal SVZ. *In situ* hybridization revealed that miR-132 expression occurs at the onset of synaptic integration in the OB. Using *in vivo* electroporation we found that sequestration of miR-132 using a sponge-based strategy led to a reduced dendritic complexity and spine density while overexpression had the opposite effects. These effects were mirrored with respective changes in the frequency of GABAergic and glutamatergic synaptic inputs reflecting altered synaptic integration. In addition, timely directed overexpression of miR-132 at the onset of synaptic integration using an inducible approach led to a significant increase in the survival of newborn neurons. These data suggest that miR-132 forms the basis of a structural plasticity program seen in SVZ-OB postnatal neurogenesis. miR-132 overexpression in transplanted neurons may thus hold promise for enhancing neuronal survival and improving the outcome of transplant therapies.

## Introduction

The adult SVZ is one of two neurogenic zones that persist in the adult brain of all mammalian species examined including humans [1], [2] (for review see [3]). The SVZ is the largest neurogenic zone and is located along the wall of the lateral ventricle beneath a layer of ependymal cells. This neurogenic region contains several cell types, including neural progenitor cells, intermediate progenitors, and neuroblasts. Neuroblasts migrate along a rostral migratory stream (RMS) to the olfactory bulb (OB) where they mature and synaptically integrate as interneurons. Identifying the molecular signals controlling the different steps of neurogenesis from neuron production to synaptic integration and survival is critical for future therapeutic strategies aimed at promoting endogenous repair and improving the success of neural transplants. A whole symphony of intracellular and extracellular signals that affect the early stages of neurogenesis (*i.e.* proliferation, fate commitment, and migration) has been identified [4], [5]. However, much less is known regarding the intracellular molecules controlling the late stages of neurogenesis (*i.e.* dendrite development, synaptic integration and survival) [4].

The cAMP response element binding protein (CREB) is a long studied transcription factor that is important for the survival and dendritic arborization of newborn OB neurons [6]. CREB controls the expression of many molecules including an activity-dependent microRNA (miR) miR-132 [7]. microRNAs are short, non-coding, single-stranded RNA molecules approximately 19–23 nucleotides in length that regulate gene expression by binding to complementary elements in the untranslated regions of target mRNAs and inhibiting protein synthesis [8]–[10]. Intriguingly, the CREB-dependent miR-132 has been shown to control the development of dendrites and spines, and synaptic integration in cultured hippocampal neurons and newborn hippocampal neurons [7], [11]–[16]. More specifically, it was reported that knockout of the miR-212/132 locus using conditional transgenic mice or knockdown of miR-132 using viral vectors led to reduced dendritic complexity and spine density, respectively, in newborn neurons of the adult hippocampal neurogenic zone [14], [16]. The dendritic effect was shown to be preferentially due to miR-132 loss.

We thus set out to investigate whether miR-132 acts in the late stages of SVZ neurogenesis using both sequestration and overexpression strategies *in vivo*. Using *in situ* hybridization we found that miR-132 expression mirrors that reported for CREB [6] and occurs at the onset of synaptic integration. Sequestration of miR-132 in newborn neurons led to a reduced dendritic complexity and spine density while overexpression had the opposite effect. In addition, timely directed overexpression at the onset of synaptic integration using an inducible approach led to a significant increase in the survival of newborn neurons. These data suggest that the CREB-regulated miRNA miR-132 forms the basis of a structural plasticity program seen in SVZ postnatal neurogenesis.

## Results

### Newborn neurons progressively express miR-132 at the onset of synaptic integration

**Figure 1:**
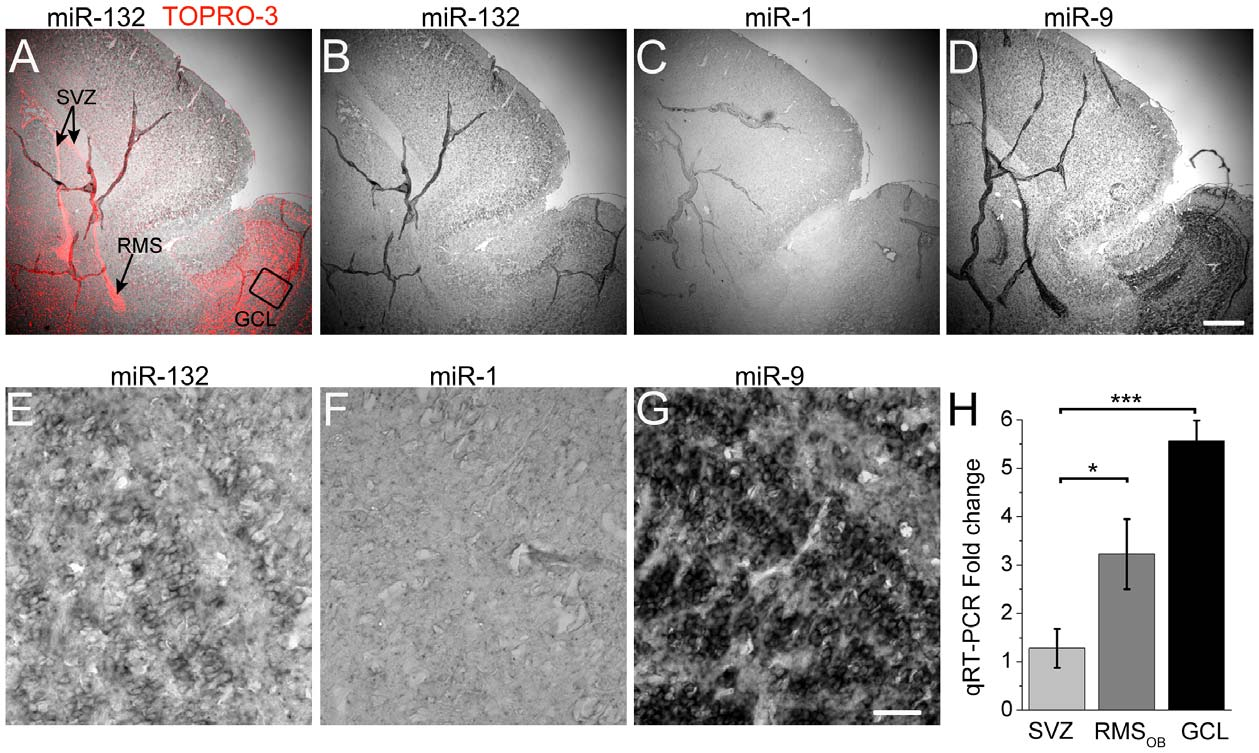
miR-132 is expressed in newborn SVZ neurons at the onset of synaptic integration. (**A–D**) *In situ* hybridization images of miR-132 with TOPRO-3 (red) overlay (red, A), miR-132 (B), miR-1 (C), and miR-9 (D) in a sagittal section containing the SVZ, RMS and OB. (**E–G**) Higher magnification of miR-132, miR-1 and miR-9 images in the granule cell layer (GCL). Scale bars: 100 µm (A–D) and 30 µm (E–F). The image in (E) comes from the boxed region in (A). (**H**) Bar graphs of miR-132 qRT-PCR fold changes in the RMS_OB_ and GCL compared to the SVZ.

To examine whether newborn neurons along the SVZ-OB axis express miR-132, we performed *in situ* hybridization in postnatal (P) 21 sagittal sections (**Figure 1**). We also examined the expression of microRNAs miR-1, which is present at very low levels in the central nervous system (CNS) and miR-9, which is enriched in developing neural regions [17]. Co-staining for the nuclear marker TOPRO-3 highlighted the SVZ, RMS, and OB due to the high cell density (**Figure 1A**). miR-132 was not expressed in the SVZ or proximal RMS, but was present in the RMS of the OB (RMS_OB_) and in the granule cell layer (GCL, **Figure 1B and 1E**). miR-132 expression intensity was lower than that of miR-9 but above miR-1 (**Figure 1B–G**). Similarly, miR-132 was expressed at a lower level than miR-9 in the hippocampal CA1 and CA3 fields and the granule cell layer of the dentate gyrus (**Figure S1**). Quantitative RT-PCR (qRT-PCR) for miR-132 from microdissected SVZ, RMS_OB_ and GCL confirmed that miR-132 expression significantly increased along the SVZ-OB axis and was 5.5-fold higher in the GCL compared to the SVZ (**Figure 1H**).

### miR-132 knockdown and sequestration truncates dendritic development leading to synaptic input deprivation

We first examined whether miR-132 function on dendritic morphogenesis was conserved in newborn OB neurons using an *in vitro* assay. To knock down miR-132, we used locked nucleic acid (LNA) oligonucleotides against miR-132 (LNA^132^) and a scrambled sequence (LNA^SCR^) in cultured OB neurons. Neurons transfected with LNA^132^ for 7 days (from day 7 to 14) had significantly less complex and shorter dendrites than LNA^SCR^ transfected neurons (p<0.05, data not shown).

**Figure 2:**
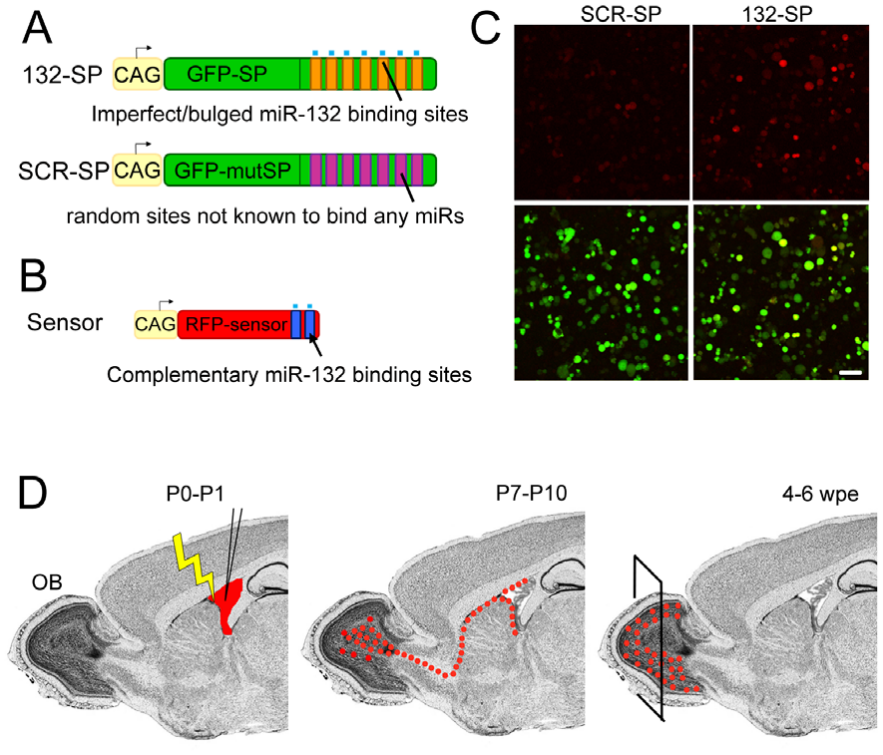
Validation of the specificity of miR-132 sponge vectors and experimental diagram. (**A**) Schematic of vectors encoding GFP containing 20 tandem, miR-132-binding sites in its 3′UTR (orange bars) to “sponge” out miR-132 (132-SP) and a control vector encoding mutant GFP containing 20 random sites in its 3′UTR (SCR9-SP). (**B**) Schematic of a sensor vector encoding RFP containing perfectly complementary miR-132 binding sites (blue bars). (**C**) Confocal images of Neuro-2A cells transfected with the sensor vector and SCR-SP or 132-SP. Scale bar: 70 µm. (**D**) Diagram of our experimental paradigm. DNA constructs were introduced into the lateral ventricle of P0–P1 pups for electroporation into neural progenitor cells. 4–6 weeks post-electroporation, fluorescently tagged, synaptically integrated newborn neurons were analyzed.

To next examine the effect of miR-132 loss-of-function on dendritic morphogenesis *in vivo*, we used a sequestration vector called “sponge” [8], [18]. Expression of mRNA constructs containing multiple (1–20) miR-132 binding sites with central mismatches in the 3′UTR of a pCAG-GFP vector (132-SP) is expected to sequester miR-132 resulting in loss-of-function and GFP expression (**Figure 2A**). Control sponges (noted SCR-SP) contained a similar number of random sites that are not known to bind any microRNA. The efficiency of these sponge constructs was validated *in vitro* using a red fluorescent protein (RFP)-based miR-132 sensor. This vector encodes RFP containing miR-132 target sites in its 3′UTR (**Figure 2B**). Each sponge vector was transfected together with the sensor in cultured Neuro-2a cells. The 132-SP vector de-repressed RFP expression while SCR-SP did not (**Figure 2C**).

**Figure 3:**
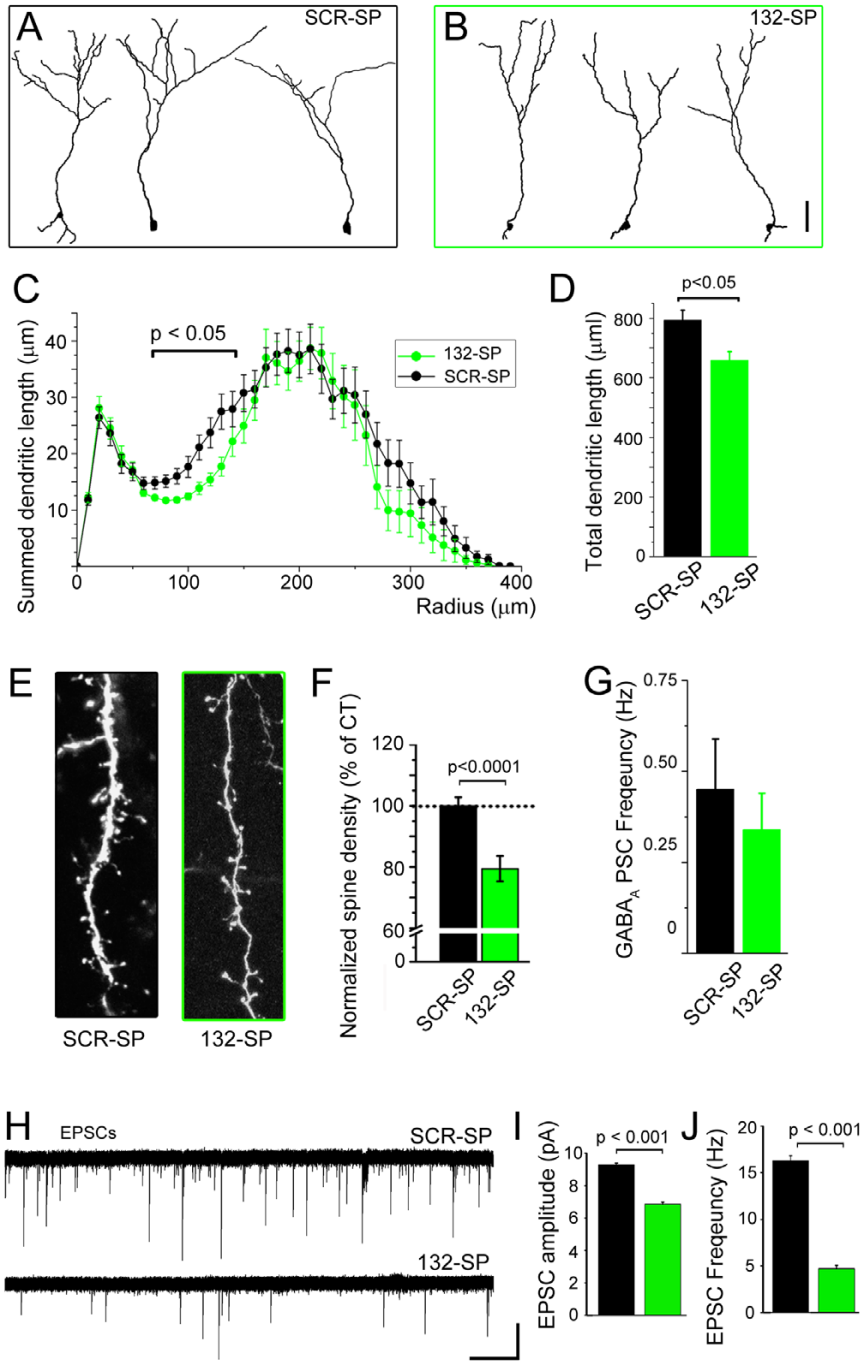
miR-132 sequestration *in vivo* truncates dendritic development leading to synaptic input deprivation. (**A and B**) Representative reconstructions of SCR-SP- (A) and 132-SP-expressing (B, green) newborn neurons at 6 wpe in the GCL. (**C and D**) Plots of the summed dendritic length (C) and bar graphs of the total dendritic length (D) of SCR-SP-(black) and 132-SP-expressing (B, green) newborn neurons (n = 22 and 25 neurons, respectively). (**E**) Confocal images of spines in fluorescent neurons containing: SCR-132 (black) or 132-SP (green). (**F**) Comparison of normalized spine density. N = 3 mice for each condition. (**G**) Bar graph of the mean frequency of GABA_A_ PSCs in SCR-SP (black) and 132-SP (green) neurons (n = 18 and 16 neurons, respectively). (**H**) Representative examples of EPSCs in neurons containing SCR-SP and 132-SP. Scale bar: 10 pA/30 s. (**I and J**) Bar graphs of the mean amplitude (I) and frequency (J) in neurons containing SCR-SP (black, n = 11 cells) and 132-SP (green, n = 11 cells). Scale bar in A–B: 50 µm; in E: 10 µm.

To selectively express the sponge-based vectors into newborn OB neurons, we used neonatal electroporation into SVZ neural progenitor cells lining the lateral ventricle [19]–[21]. These neural progenitor cells generate neurons that migrate to the OB via the RMS and are synaptically mature by ∼4 weeks after birth [22]. The sponge vectors were electroporated at P0–P1 resulting in labeling of neuroblasts born during the first week post-electroporation (wpe) because of a progressive dilution of the plasmid following successive cell division (**Figure 2D**). At 4–6 wpe, the dendritic morphology, spine density, and synaptic inputs of GFP^+^ neurons were assessed in the GCL of the OB using NeuroLucida reconstruction and patch clamp recordings, respectively (**Figure 3**).

Newborn neurons expressing 132-SP displayed a significant decrease in their dendritic complexity and length compared to neurons containing SCR-SP vector at 6 wpe (p<0.01, unpaired t-test, n = 22 versus 25 neurons in control, N = 4 OB each, **Figure 3A–C**). The total length was significantly reduced by ∼13% (p<0.05, **Figure 3D**). Sequestering miR-132 significantly decreased spine density by 21% (p<0.0001, N = 3 OB each, **Figure 3E and F**).

Decreased dendritic length and spine density led us to predict that the frequency of GABAergic and glutamatergic synaptic inputs should be reduced. In the absence of glutamatergic blockers, synaptic currents recorded in the whole cell configuration were identified as GABAergic by their slow decay kinetics (6–40 ms) and their sensitivity to a GABA_A_ receptor antagonist, bicuculline (10 µM) (n = 8, data not shown). The frequency of spontaneous GABAergic postsynaptic synaptic currents (GABA_A_ PSCs) at 4–6 wpe was decreased by 24% in 132-SP-expressing neurons, but this was not significant (p = 0.28, SCR-SP: −0.45±0.14, n = 18, N = 6, vs. 132-SP: 0.34±0.10 Hz, n = 16, N = 5, **Figure 3G**). The amplitude of PSCs was not significantly different (SCR-SP: −69.1±6.2 pA, n = 18, vs. 132-SP: −70.5±9.6 pA, n = 16, data not shown). Excitatory postsynaptic currents (EPSCs) were recorded in the presence of a GABA_A_ receptor blocker (20 µM picrotoxin) otherwise EPSCs were partially masked by GABAergic activity. miR-132 sequestration led to a significant decrease in the frequency and amplitude of EPSCs compared to control (n = 11 cells each, N = 4, **Figure 3H–I**). Collectively, these data show that miR-132 acquisition at the onset of synaptic integration is important for newborn neuron dendritic morphogenesis and proper synaptic integration.

### miR-132 overexpression promotes dendritic morphogenesis and synaptic integration *in vivo*

**Figure 4:**
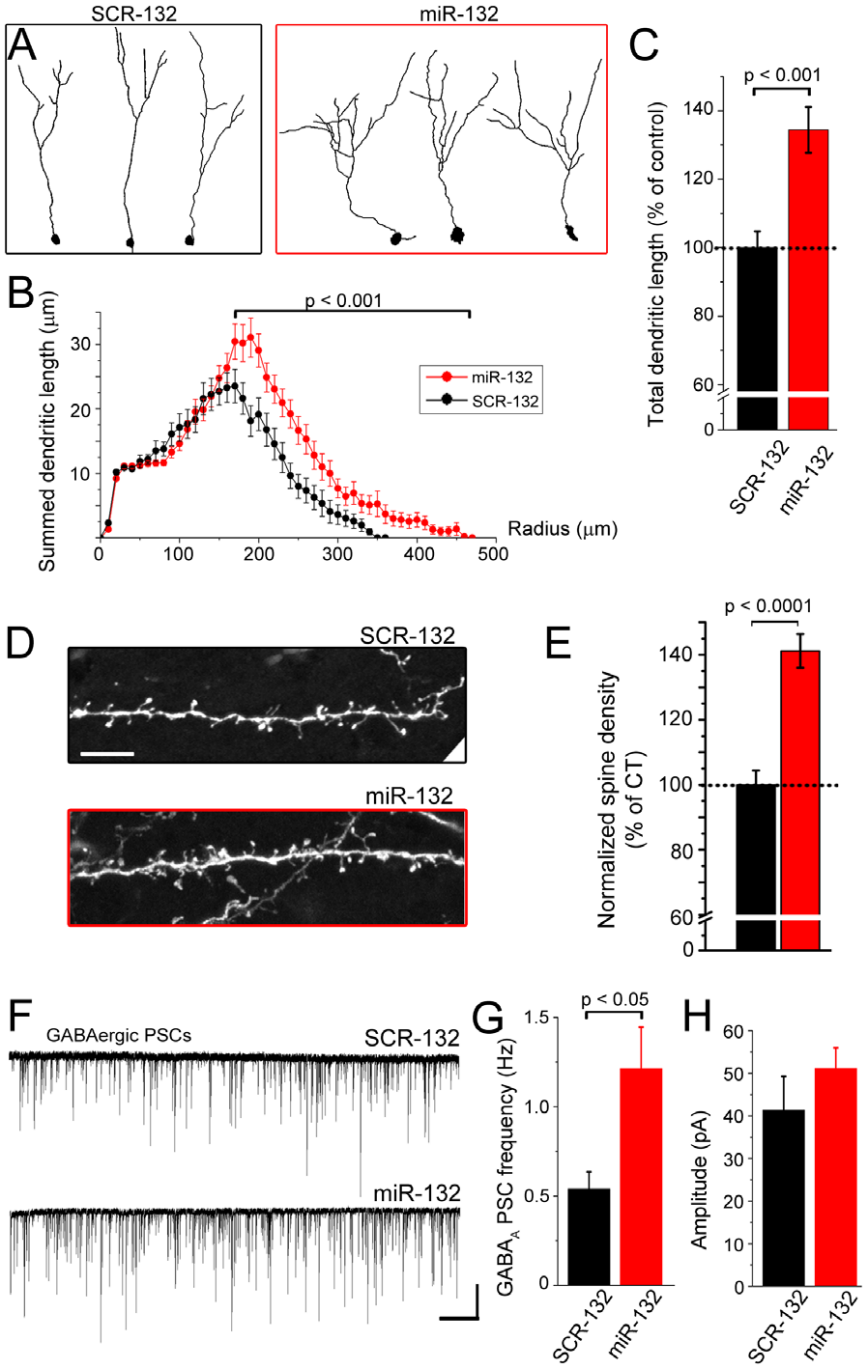
miR-132 overexpression promotes dendritic morphogenesis and synaptic integration *in vivo*. (**A and B**) Representative reconstructions of SCR-132 (A) and miR-132-expressing (B, red) newborn neurons at 8 wpe in the GCL. **(B)** Plots of the summed dendritic length of SCR-132-(black) and miR-132 expressing (B, red) newborn neurons (n = 38 and 55 neurons, respectively). **(C)** Bar graphs of the percentage (%) of control for the total dendritic length of miR-132 overexpressing neurons (red). A break in the Y-axis was inserted between 5 and 60 µm. ). **(D)** Confocal images of spines in fluorescent neurons containing: SCR-132 (black) or miR-132 (red). **(E)** Bar graphs of the normalized spine density. N = 3 mice for each condition. **(F)** Representative traces of GABAergic postsynaptic synaptic currents (PSCs) in SCR-132- and miR-132-containing neurons. **(G and H)** Bar graphs of the frequency (E) and amplitude (F) of GABAergic PSCs in SCR-132- and miR-132-containing neurons (n = 10 black and 15 red, respectively). Scale bar: 100 pA/500 ms in F.

*In situ* hybridization data show that miR-132 expression levels are lower than that of miR-9 suggesting that miR-132 levels are not saturated. We thus examined whether overexpression of miR-132 would have opposite effects to those of miR-132 sequestration. For studying miR-132 gain-of-function, a plasmid encoding miR-132 under the control of a U6 promoter and a RFP under the cytomegalovirus early enhancer element and chicken β-actin (CAG) promoter was electroporated into SVZ cells at P1. A vector containing a non-coding, scrambled sequence was used as control (SCR-132) in littermate mice. Effective miR-132 overexpression with the miR vector was validated by quantitative (q) RT-PCR *in vivo* and the RFP-based miR-sensor *in vitro*. The qRT-PCR levels of miR-132 were 4.5-fold higher in the ipsilateral OB containing RFP^+^ neurons compared to the contralateral OB (p<0.005, **Figure S2A and B**). Transfection of the miR-132-encoding vector (without a CAG-RFP sequence) together with the RFP sensor and a cyan fluorescent protein (CFP)-encoding reporter vector silenced RFP expression (**Figure S2C and D**). At 8 wpe the dendritic morphology, and at 4–6 wpe the spine density and the GABAergic synaptic inputs of GFP^+^ neurons were assessed in the GCL (**Figure 4**).

At 8 wpe, newborn neurons overexpressing miR-132 displayed a significant increase in the dendritic complexity and length compared to neurons containing a control vector (p<0.01, **Figure 4A and B**). The total length was increased by ∼35% (p<0.001, red, **Figure 4C**), which was in the opposite direction compared to the miR-132 sponge (−13%, green in Figure 3D). At 6 wpe, miR-132 overexpression increased spine density by 44% (N = 3 each, p<0.0001, **Figure 4D and E**). Consistent with increased dendritic complexity and length, the frequency of spontaneous GABAergic PSCs at 4–6 wpe was significantly increased by 124% in miR-132 overexpressor containing neurons compared to neurons containing a control vector (p<0.05, n = 10 neurons, N = 3 with miR-132 and n = 15 neurons, N = 4 with control, **Figure 4F and G**). GABAergic current amplitude tended to be larger but the change was not significant (p = 0.1, **Figure 4H**).

Collectively, these data show that miR-132 overexpression in newborn neurons enhances dendrite development resulting in stronger synaptic integration. These data also suggest that endogenous miR-132 expression is not saturated and can be increased enhancing synaptic integration of newborn neurons.

### miR-132 overexpression during synaptic integration promotes long-term neuronal survival

At 5–6 weeks after birth, half of the newborn neurons die through apoptosis [22]. Considering that miR-132 overexpression enhances synaptic integration of newborn neurons, we hypothesized that such manipulation during synaptic integration would promote long-term survival of miR-132-overexpressing neurons. An opposite effect is expected with miR-132 sequestration. The density of RFP^+^ neurons was thus assessed at 6 wpe in the OB under the two miR-132 experimental conditions. miR-132 sequestration had no effect on GCL cell density (n = 9 mice each, data not shown). Unexpectedly, miR-132 overexpression led to a significant 34% decrease in the number of miR-132-overexpressing neurons in the GCL compared to control (p<0.01, **Figure S3A**). There was no difference in the density of RFP^+^ cells in the SVZ at 8 days post-electroporation (dpe) and 6 wpe (data not shown), suggesting that the electroporation efficiency was identical between the two experimental conditions. Considering that miR-132 was overexpressed ectopically in neuroblasts at the time of their birth, we speculated that this premature overexpression triggered apoptosis. Indeed, there was a significant 8-fold increase in the density of miR-132-overexpressing neuroblasts that were positive for activated caspase-3 compared to control neuroblasts in the RMS_OB_ at 8 dpe (p<0.05, **Figure S3B and E**). This latter effect presumably reflected a premature maturation of neuroblasts in the RMS leading to apoptosis in the absence of proper survival cues.

To circumvent cell death, we used an inducible Cre-Lox based plasmid vector to overexpress miR-132 at a time matching its physiological acquisition. The vector, called pSico (*i.e.* plasmid for stable RNA interference conditional [23]) contains a U6 promoter followed by LoxP sites around a CMV promoter driving GFP acting as a Stop sequence prior to the miR-132- or the SCR-132 sequence. Following co-electroporation of pSico and a vector encoding ^ERT2^Cre^ERT2^ (and a RFP-encoding reporter vector), subcutaneous tamoxifen applications are expected to allow timed miR-132 expression in pSico-containing neurons. Based on the *in situ* data in Figure 1, neurons express miR-132 at the time of entry in the OB, which occurs at ∼7–10 dpe. Tamoxifen was thus administered at P7. Two injections led to the loss of GFP in RFP^+^ neurons suggesting that the Stop sequence was properly excised (n = 5 animals, data not shown). In the absence of ^ERT2^Cre^ERT2^ co-electroporation, RFP^+^ cells were GFP^+^ (n = 3 animals, data not shown).

**Figure 5:**
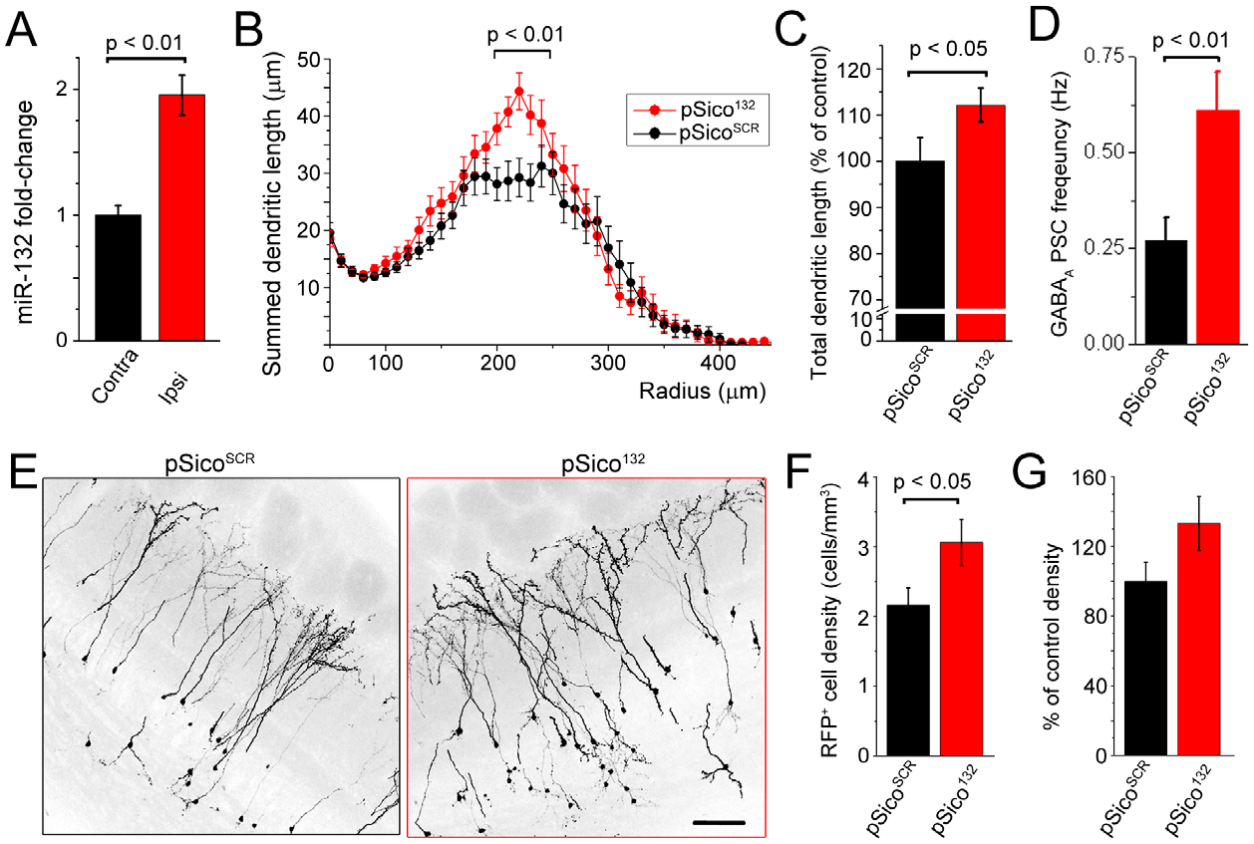
miR-132 overexpression at synaptic integration promotes long-term neuronal survival. (**A**) qRT-PCR of miR-132 fold-change normalized to control RNA U6 from the ipsilateral bulbs containing pSico^132^-expressing neurons (red) and from the contralateral bulbs (black) at 5 weeks post-tamoxifen (wpt) injections given 2 wpe (N = 5 mice each). (**B and C**) Plots of the summed dendrite length (B) and bar graphs of the total dendritic length (C) of pSico^SCR^ (n = 47) and pSico^132^-containing neurons (n = 51) at 5 wpt injections given at 7 dpe (i.e. 6 wpe). (**D**) Bar graphs of the frequency of GABAergic PSCs in pSico^SCR^- and pSico^132^-containing neurons at 5 wpt (n = 13 black and 15 red, respectively). (**G**) Sample images illustrating the density of pSico^SCR^ and pSico^132^ neurons in OB coronal sections. (**H and I**) Bar graphs of absolute (H) and normalized (I) RFP^+^ (*i.e.* pSico^SCR^, black and pSico^132^, red) neuron density in the GCL (N = 8 and 9 mice, 3–4 images per mouse, respectively). Scale bar: 100 µm.

The efficiency of this strategy was validated using qRT-PCR for miR-132 from ipsi- and contra-lateral OB 5 weeks following tamoxifen injection (**Figure 5A**). At 6 wpe (*i.e.* 5 weeks post-tamoxifen), pSico^132^-neurons displayed significantly enhanced dendritic complexity and length (p<0.01, n = 47 control and 51 pSico^132^, N = 4 and 5, **Figure 5B and C**) and increased frequency of GABA_A_ PSCs compared to pSico^SCR^-neurons (p<0.01, n = 13 control and 15 pSico, N = 4 each, **Figure 5D**). Importantly, there was a significant 33% increase in the number of pSico^132^-neurons that integrated in the OB compared to pSico^SCR^-neurons (p<0.05, **Figure 5E–G**).

Collectively, these data strongly suggest that miR-132 expression at the entry into a synaptic network enhances synaptic integration and long-term survival of newborn neurons.

## Discussion

Here, we show that miR-132 is involved in the morphological development and synaptic integration of neurons arising from the neonatal neurogenic SVZ. In addition, timed overexpression of miR-132 in newborn neurons at the onset of synaptic integration significantly enhanced the strength of their connections and their long-term survival. Since expression of miR-132 is activity-dependent, these findings suggest that miR-132 may function as an intrinsic effector of activity-dependent processes in newborn neurons, coupling incoming information and environmental cues to synaptic integration.

Data from *in situ* hybridizations and qRT-PCR indicate that newborn neurons express miR-132 at the onset of radial migration in the OB, which coincides with their synaptic integration. Regarding miR-9, its level was higher than miR-132 all along the SVZ-RMS axis suggesting that it plays an important role in regulating postnatal neurogenesis. In fact, miR-9 has been shown to regulate neurogenesis in the mouse telencephalon, and in particular cell proliferation and differentiation [24]. The role of miR-9 on SVZ neurogenesis remains to be explored. Here, finding miR-132 expression at the onset of synaptic integration is in agreement with the reported expression of CREB, which controls miR-132 expression [6], [7]. The levels of mature miR-132 transcript are not basally high in neurons, including newborn SVZ-OB neurons. This finding fits with the activity-dependence of pre-miR-132 transcript and miR-132 expression [25], [26] and our data showing that miR-132 levels are not saturated.

Our results demonstrate a role for miR-132 in the late stages of SVZ neurogenesis. Another micro-RNA miR-124 had been reported to regulate an early stage of SVZ neurogenesis (fate commitment) [27]. Here, miR-132 overexpression increased morphological complexity, the total dendritic length, spine density, and the frequency of GABAergic postsynaptic currents. Dendritic morphology was the only parameter analyzed at 8 wpe, but a similar increase in dendritogenesis is expected at 6 wpe considering the marked effect on the frequency of GABAergic synaptic activity. By contrast, miR-132 sequestration decreased morphological complexity, dendritic length, spine density, and the frequency of glutamatergic synaptic currents at 4–6 wpe. Although not significant, there was a trend of decreasing frequency of GABAergic currents by sequestration of miR-132. This may be secondary to the fact that overexpression causes a 35% increase in dendritic length, but there was only a 13% decrease by sequestration. These findings are in agreement with previous reports *in vitro* and *in vivo*. miR-132 knockdown or overexpression decreased and increased morphological complexity and spine density, respectively [7], [11]–[16]. We have not identified a miR-132 target regulating dendritic morphogenesis, but a previous study reported p250GAP as a miR-132 target that mediated its effect on dendritic plasticity [15]. Sponging miR-132 did not result in any significant change in long-term survival of newborn neurons again presumably because the effect of the sponge was mild on neuronal morphology and synaptic activity. miR-132 overexpression in newborn neurons at birth does not increase the number of integrating cells or their long-term survival, but rather induces apoptosis. The simplest explanation is that improperly timed or extended overexpression of miR-132 and thus synaptic integration-related genes is toxic for neurons, and the increase in programmed cell death that we see may be explained as an “excitotoxic” effect of prolonged miR-132 overexpression. To circumvent this problem, we designed an inducible miR-132 vector allowing timed expression of miR-132 at the time of synaptic integration. This powerful strategy revealed that such overexpression of miR-132 led to the acquisition of more complex dendritic tree, more synaptic connections cumulating in enhanced long-term survival.

Collectively, our findings show that miR-132 orchestrates a structural plasticity program during SVZ neurogenesis *in vivo*. For the first time, our data show a bidirectional role for miR-132 in regulating dendritic morphogenesis and synaptic physiology using novel strategies *in vivo*. Importantly, using an inducible Cre-Lox strategy, which can be applied to other systems, we show that miR-132 overexpression during a critical period of synaptic integration enhanced long-term survival of newborn neurons. This finding raises the possibility that inducible miR-132 expression in transplanted neurons using selective promoter driving Cre may be used to enhance neuronal survival and transplant efficiency.

## Materials and Methods

### Mice

Animal protocols were approved by the Yale University Institutional Animal Care and Use Committee. All experiments were performed in P0 to P56 CD1 mice (Charles River Laboratories, MA).

### microRNA in situ hybridization

Mice were deeply anesthetized via isoflurane inhalation and transcardially perfused with saline followed by 4% paraformaldehyde (both ice-cold). Brain tissue was dissected out, equilibrated in 30% sucrose, then embedded in OCT (Tissue Tek) and frozen in liquid nitrogen-cooled isopentane. 10 µm thick serial cryosections were cut in the sagittal plane on a Leica CM3050S cryomicrotome, mounted on Superfrost Plus slides and stored at −20°C until hybridization could begin. In situ hybridization was carried out as outlined previously with a few modifications [28]. Slide-mounted sections were air-dried, then re-fixed in 4% paraformaldehyde for 10 minutes at room temperature. Following rinsing in DEPC-treated PBS slides were acetylated in acetylation solution (590 ml DEPC water, 8 ml triethanolamine, 1050 µl 37% HCl and 1.5 ml acetic anhydride) for 10 minutes with gentle agitation, and then washed in DEPC-PBS once for 5 minutes without agitation. Next, slides were treated with Proteinase K (5 µg/ml in DEPC-PBS) for 5 minutes. Slides were washed twice in DEPC-PBS again and then prehybridized by pipetting 700 µl prehybridization solution (50% formamide, 5× SSC, 5× Denhardt's, 200 µg/ml yeast RNA, 500 µg/ml salmon sperm DNA, 0.4 g Roche blocking reagent and 1.75 ml DEPC water) onto sections and covering with parafilm for 4–8 hours at room temperature. For hybridization 1 µl of 25 µM digoixigenin (DIG) or fluorescein isothiocyanate (FITC) double-labeled LNA probe was added to 1000 µL hybridization buffer (same as prehybdrization buffer but with 500 µl 10% CHAPS, 100 µl 20% Tween and 1.15 ml DEPC water). Probes were denatured at 80°C for 5 minutes and then kept on ice. 150 µl of the hybridization buffer and probe was applied to the tissue sections. Sections were then cover-slipped with RNAse-free plastic coverslips and gently rocked for 12–16 hours at 50–60°C (∼20°C below the predicted melting temperature (Tm) of probe:miRNA) in a humidified chamber. Following hybridization, coverslips were removed in pre-warmed 5× SSC at 60°C. Slides were then washed in warm 0.2× SSC for 1 hour at 60°C, followed by buffer B1 (0.1 M Tris pH 7.5/0.15 M NaCl) for 10 minutes at room temperature. Sections were then blocked in 10% fetal calf serum made up in B1 buffer for 1 hour at room temperature and probed with anti-DIG/FITC antibodies conjugated to alkaline phosphatase (Southern Biotech) at a 1∶2000 dilution overnight at 4°C. Following incubation with primary antibodies slides were washed in B1 buffer thrice and then equilibrated in buffer B3 (0.1 M Tris pH 9.5/0.1 M NaCl/50 mM MgCl_2_) for 10 minutes. Developer solution (100 mg/ml NBT, 50 mg/ml BCIP, 24 mg/ml levamisol and 10% Tween in B3) was then added to the tissue for ∼4 hours at room temperature. The color reaction was stopped with washes in PBT, and sections were then incubated with DNA dye TO-PRO-3 iodide (Invitrogen) for 10 minutes. Following final washing steps slides were mounted in Aquamount and visualized using confocal microscopy.

### qRT-PCR

RNA enriched in small RNAs was isolated from tissue samples and cultured cells using the miRVana RNA extraction kit (Ambion). Mature miRNA expression was assayed using Applied Biosystems' individual TaqMan microRNA assays that include primers for both the reverse transcription and real-time PCR reactions. These reactions were carried out according to protocols provided by the vendor. Briefly, 15 µl reverse transcription reactions consisted of 10 ng total RNA, 1× TaqMan miRNA reverse transcription primer, 1.0 mM of each dNTP, 50.0 U MuLV Reverse Transcriptase, 1× Reverse Transcription Buffer, 0.25 U/µl RNase Inhibitor and nuclease-free water. Reverse transcription reactions were incubated at 16°C for 30 min, 42°C for 30 min, and 85°C for 5 min. Real-time PCR reactions were performed on an Applied Biosystems 7900HT SDS platform. PCR reactions were carried out in triplicate for each sample. 10 µl PCR reactions consisted of 1× TaqMan Universal Master Mix, No AmpErase UNG, 1× TaqMan miRNA assay mix, 1.33 µl reverse-transcribed cDNA and nuclease-free water. PCR reactions were incubated at 95°C for 10 min, followed by 40 cycles of 95°C for 15 sec, and 60°C for 1 min (a standard protocol offered within SDS software). miRNA relative quantities (RQ) were determined using the ΔCt method and the small RNA U6 (RNU6B) was used as an endogenous control.

### LNA and Vectors

LNA for miR-132 were purchased from Exiqon. For sponge experiments, imperfect miR-132 binding sites were repeated up to 20 times in the 3′UTR of a pCAG-GFP vector (called 132-SP). The control vector repeated this strategy with a scrambled binding site (SCR) not known to correspond to any known miR (SCR-SP). For miR overexpression experiments, a mature miR-132 expressing sequence was inserted downstream from the U6 RNA promoter and into a vector co-expressing RFP downstream from a CAG, thus yielding pCAG-RFP::U6-miR-132 (noted miR-132 vector). A scrambled sequence (SCR) was used to generate the control non-coding vector pCAG-RFP::U6-SCR-132. For inducible overexpression, the Cre-Lox conditional vector, pSico (Addgene, M. Jacks) was used to place the miR-encoding sequence behind the floxed-GFP cassette. The pSico plasmids were co-injected with the tamoxifen-inducible Cre-recombinase expression vector pCAG-^ERT2^Cre^ERT2^ (Addgene, C. Cepko) as well as a pCAG-tdTomato (noted RFP) vector that was constructed using the pCMV-tdTomato vector from Clontech. Upon inducing ^ERT2^Cre^ERT2^-recombinase activity with tamoxifen, the EGFP cassette would recombine out and place the miR-encoding region directly downstream from the U6 RNA promoter (pSico^132^). The control vector repeated this strategy with a scrambled (SCR) sequence (pSico^SCR^). A miR-132 sensor vector was constructed by introducing miR-132 binding sites into the dual-fluorescent GFP-reporter/mRFP-sensor (a kind gift from Dr. De Pietri Tonelli, Neuroscience and Brain Technologies, Italy [29]), after removing the GFP-encoding sequence and placing the RFP-sensor downstream from CAG.

### LNA transfection in primary neurons and Neuro-2a cells

Olfactory bulbs from P0–P1 pups were dissected out and placed in chilled Hibernate-E (Brainbits) supplemented with 2% B-27 (Invitrogen) and 0.5 mM GlutaMAX (Gibco). Tissue was dissociated by incubating in papain and with mechanical trituration as indicated in the Neural Tissue Dissociation Kit (Miltenyi Biotec). The cell suspension was then separated on a density gradient using OptiPrep (density 1.32). The neuronal fraction was collected, washed, and re-pelleted in Hibernate-E/B-27/GlutaMAX. The Neuro-2a mouse neuroblastoma cell line (American Type Culture Collection) was routinely propagated in tissue culture treated polystyrene multi-well plates or flasks (BD Falcon). For both primary neurons and Neuro-2a cells, the medium consisted of Dulbecco's Modified Eagle Medium (Invitrogen) supplemented with 10% heat-inactivated fetal calf serum and penicillin-streptomycin at 100 U/l and 100 µg/l each (Gibco). Cells were maintained at 37°C and 5% CO_2_. For LNA and miR overexpression assays, LipofectAMINE 2000 (Gibco) was used to transfect expression vectors according to the manufacturer's instructions. Transfection efficiency was verified by examining the green fluorescence of LNA or the fluorescence from other vectors (see above). For Neuro2A cells, cells were examined 96 hrs post-transfection.

### Immunohistochemistry

6–8 weeks following electroporation, mice were deeply anesthetized via isoflurane inhalation and transcardially perfused with saline followed by 4% paraformaldehyde. Brain tissue was dissected out, equilibrated in 30% sucrose, then embedded in OCT (Tissue Tek) and sectioned into 100 µm-thick serial cryosections on a freezing sliding microtome in the coronal plane. Sections were stored in antifreeze (500 ml 0.1 M Tris-buffered saline, 300 ml ethylene glycol, 300 g sucrose, 10 g polyvinylpyrrolidone and distilled H_2_O to a volume of 1 L) at −20°C until immunostaining could begin. Slices were washed several times in phosphate buffered saline (PBS), then incubated in blocking buffer (2% horse serum, 1% bovine serum albumin, 0.1% Triton-X, 0.1% Tween-20 in PBS) for 1 hour at room temperature. Slices were then incubated in blocking buffer containing the following antibodies at 4°C overnight: rabbit anti-RFP (1∶500, Rockland Immunochemicals), rat anti-RFP (1∶500, Chromotek), and chicken anti-GFP (1∶500, Abcam), rabbit anti-activated caspase 3 (1∶500, Cell signaling). After several washes the next day slices were incubated with an appropriate secondary antibody (Alexa Fluor dye conjugates at 1∶1000, Invitrogen; and Cyanine and DyLight dye conjugates at 1∶500, Jackson ImmunoResearch) for 1 hour at room temperature. Sections were then washed again several times, incubated in the DNA dye TOPRO-3 iodide and mounted in Prolong Gold antifade reagent (Invitrogen). Images were acquired using an Olympus Fluoview 1000 confocal microscope (optical section step size 2 µm with a 20× objective, numerical aperture 0.75, and 0.5–1 µm with a 60× oil immersion objective, numerical aperture 1.40).

### Neonatal *in vivo* electroporation

Plasmids (2–5 µg/µl) were diluted in PBS containing 0.1% fast green as a tracer. 0.5–1 µl of plasmid solution was injected into the lateral ventricles of cold-anesthetized neonatal pups, using a pulled glass pipette beveled to a diameter of less than 50 µm. After plasmid injection using manual pressure, tweezer-type electrodes (model 520, BTX) were placed on the heads of the P0–P2 pups and 4 square-pulses of 50 ms duration with 950 ms intervals at 135 V were applied using a pulse ECM830 BTX generator. Pups were recovered with gentle heat and reunited with the mother.

### Morphometry

Plasmid-expressing OB granule neurons of the superficial GCL were identified in coronal sections using RFP fluorescence. Similarly in vitro, LNA-expressing neurons were identified using green fluorescence. Complete neurons in confocal z-stacks acquired at 20× were traced using the NeuroLucida and NeuroExplorer morphometry software (MicroBrightField). Sholl analyses were blindly carried out using dendrite length as a measure of morphological complexity. Confocal z-stacks from 3 different square fields of view were taken from each OB section, and this was done for 3 different OB sections in a randomly selected series from each animal. At least 3 animals were analyzed per condition.

Spine analysis was carried out on a series of sequentially acquired coronal olfactory bulb slices, from 3 animals per condition. Confocal z-stacks of dendritic segments in the external plexiform layer were acquired at 60× with a 0.5 µm-step size, blindly traced using NeuroLucida and NeuroExplorer morphometry software.

### Electrophysiology

Horizontal OB slices (300 µm) were prepared from anesthetized (Nembutal 100 mg/kg, intraperitoneal) P28 to P42 mice using a Leica VT1000S vibratome (Nussloch, Germany). An artificial CSF (ACSF) dissection solution with reduced Ca^2+^ contained the following (in mM): 124 NaCl, 2.6 KCl, 1.23 NaH_2_PO_4_, 3 MgSO_4_, 26 NaHCO_3_, 10 dextrose, and 1 CaCl_2_, equilibrated with 95% O_2_/5% CO_2_ and chilled to 4°C during slicing. Brain slices were incubated in a 30°C water-bath for 30 min and then maintained at room temperature. During experiments, slices were superfused with ACSF at room temperature that contained the following (in mM): 124 NaCl, 3 KCl, 1.23 NaH_2_PO_4_, 1.2 MgSO_4_, 26 NaHCO_3_, 10 dextrose, and 2.5 CaCl_2_, equilibrated with 95% O_2_/5% CO_2_. Whole-cell patch-clamp recordings were made from fluorescent OB granule cells that contained the vector of interest. Patch electrodes (5–7 MΩ resistance) contained the following for recording GABAergic PSCs (in mM): 135 KCL, 1 NaCl, 10 HEPES, 0.2 EGTA, 2 MgATP, 0.2 NaGTP. For recording EPSCs, the intracellular solution containing (in mM): 125 KGluconate, 10 KCl, 1 NaCl, 10 HEPES, 0.2 EGTA, 2 MgATP, 0.2 NaGTP. pH adjusted to 7.4 by KOH. Osmolarity was adjusted to 280–290 mOsm using a Wescor 5500 vapor pressure osmometer (Logan, UT). PSCs were analyzed using Synaptosoft's MiniAnalysis Program (Fort Lee, NJ). At least 10 minutes of recordings were obtained for each neuron and the entirety of the file was analyzed for events. The average amplitude and frequency were obtained for each neuron and compared between test and control conditions. The recordings of GABAergic synaptic currents were performed without adding glutamatergic blockers because they were easily detected from glutamatergic synaptic currents. Glutamatergic synaptic currents were recorded in the presence of the GABA_A_ receptor blocker picrotoxin, otherwise they were partially masked by the GABAergic activity.

### Cell density measurement

For cell density measurements confocal z-stacks of serial, coronal sections were acquired at 10× (numerical aperture 0.45). ImageJ (NIH) was used to manually count cells in a given volume to calculate cell density. All cells were counted in 3 different OB sections in a randomly selected series from each animal. At least 3 animals were analyzed per condition.

### Tamoxifen experiments

In the conditional overexpressor condition, tamoxifen was subcutaneously delivered at 100 µg/g to pups 7 days following electroporation, 2 injections, 4 hours apart.

### Statistical analysis

Statistical significance was determined using the two-tailed Student's t test (p<0.05) unless otherwise noted. Data are presented as mean ± standard error of the mean (SEM).

## Supporting Information

Figure S1 **miR-132 is expressed in hippocampal neurons.** (**A–D**) *In situ* hybridization images of miR-132 with TOPRO-3 (red) overlay (red, A), miR-132 (B), miR-1 (C), and miR-9 (D) in a sagittal section containing the hippocampus. (**E–G**) Higher magnification of miR-132, miR-1 and miR-9 images in the dentate dyrus. Scale bars: 100 µm (A–D) and 30 µm (E–F).
(PDF)

Figure S2 **Validation of the efficiency and specificity of miR-132 overexpression vectors.** (**A**) Diagram of the ipsilateral (ipsi) OB containing RFP^+^ neurons and contralateral (contra) OB. (**B**) qRT-PCR of miR-132 fold-change normalized to control RNA U6 from the ipsilateral OB containing miR-132-overexpressing neurons (red) and from the contralateral OB (black,  = 3 OB each). (**C**) Schematic of the miR-132 overexpression or scramble vectors (after RFP sequence removal), the RFP-based sensor vector and the CFP reporter vector. (**D**) Confocal images of Neuro-2A cells transfected with the sensor vector, the CFP reporter vector and either SCR-132 or miR-132. Scale bars: 70 µm.
(PDF)

Figure S3 **miR-132 overexpression in neuroblasts at birth led to apoptosis.** (**A**) Bar graphs of the RFP^+^ (*i.e.* SCR-132, black and miR-132, red) neuron density in the GCL at 6 wpe (N = 3 mice each, 3–4 images analyzed per mouse). (**B**) Bar graphs of the percentage of RFP^+^ neuron being activated Caspase 3-positive (Casp3^+^) in the RMS_OB_ at 8 dpe (N = 3 mice each, respectively). (**C–F**) Sample images of Casp3 staining (green) with TOPRO-3 (blue, C and E) and RFP staining (red) with TOPRO-3 (blue, D and F) in the RMS_OB_ containing SCR-132 (C and D) and miR-132 (E and F) -expressing newborn neurons. Scale bar: 100 µm.
(PDF)
