# Supplementary figures and images for: miR-132 Enhances Dendritic Morphogenesis, Spine Density, Synaptic Integration, and Survival of Newborn Olfactory Bulb Neurons
Source: PLoS One. 2012 May 31;7(5):e38174. doi: 10.1371/journal.pone.0038174 (PMC3364964; doi:10.1371/journal.pone.0038174)

Figure S1, Pathania

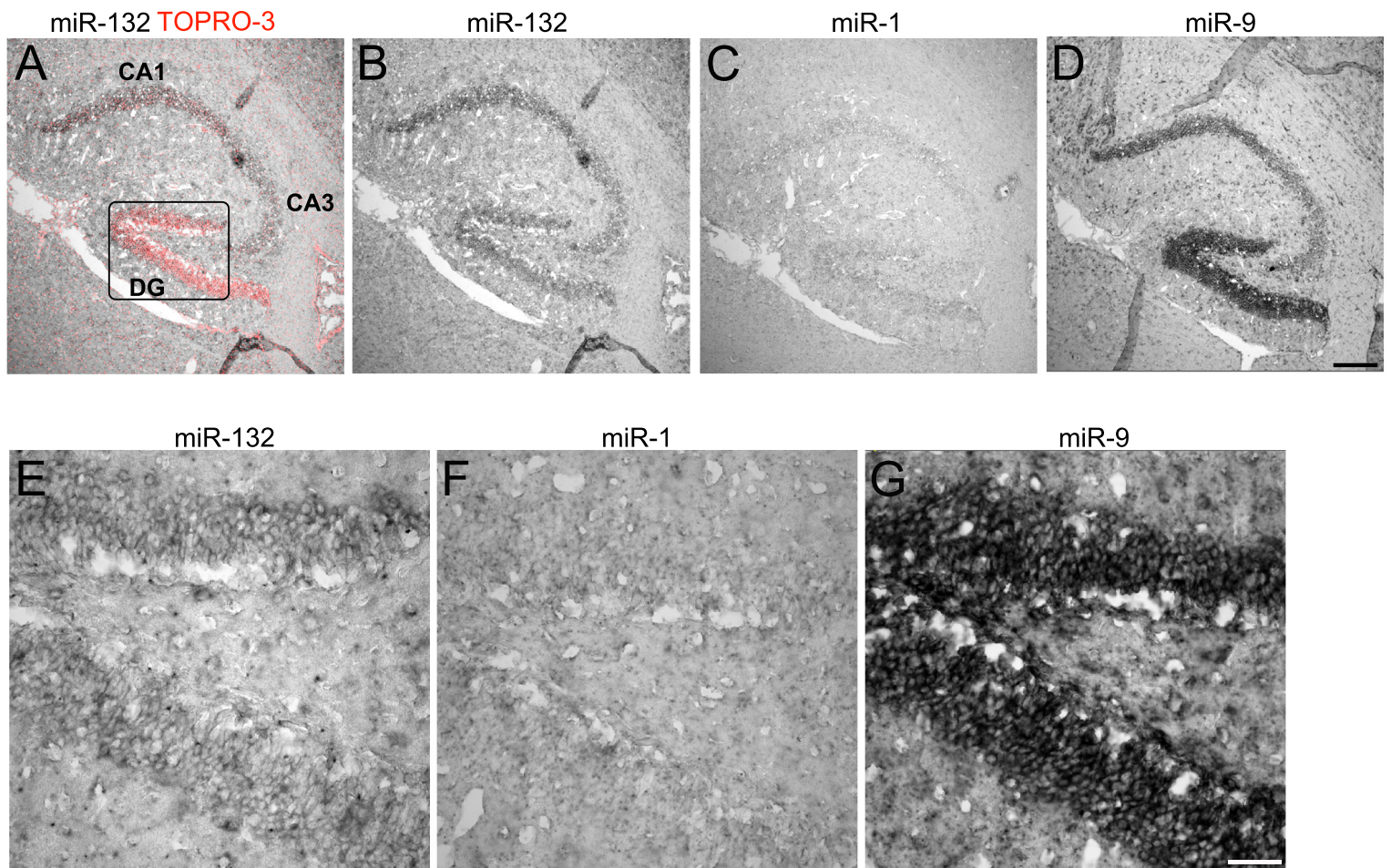

Supplement: Figure S1 — miR-132 is expressed in hippocampal neurons. (A–D) In situ hybridization images of miR-132 with TOPRO-3 (red) overlay (red, A), miR-132 (B), miR-1 (C), and miR-9 (D) in a sagittal section containing the hippocampus. (E–G) Higher magnification of miR-132, miR-1 and miR-9 images in the dentate dyrus. Scale bars: 100 µm (A–D) and 30 µm (E–F). (PDF) [file pone.0038174.s001.pdf]

Figure S2, Pathania

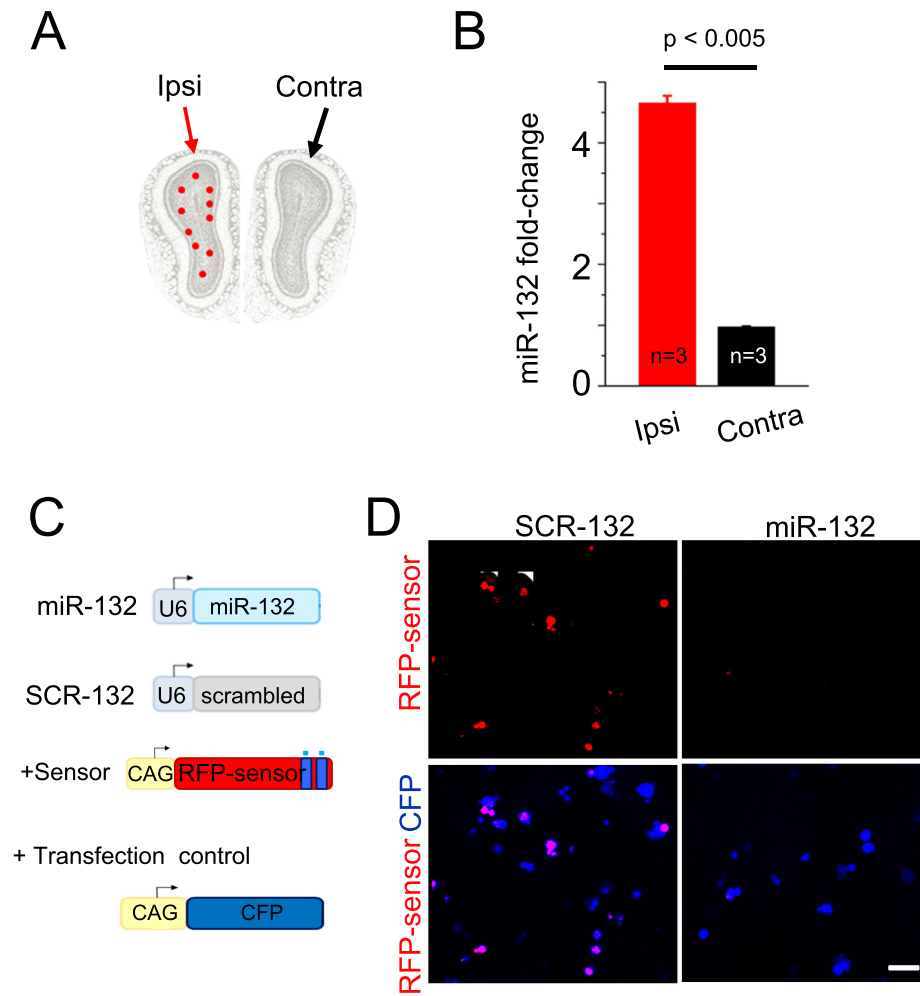

Supplement: Figure S2 — Validation of the efficiency and specificity of miR-132 overexpression vectors. (A) Diagram of the ipsilateral (ipsi) OB containing RFP+ neurons and contralateral (contra) OB. (B) qRT-PCR of miR-132 fold-change normalized to control RNA U6 from the ipsilateral OB containing miR-132-overexpressing neurons (red) and from the contralateral OB (black, = 3 OB each). (C) Schematic of the miR-132 overexpression or scramble vectors (after RFP sequence removal), the RFP-based sensor vector and the CFP reporter vector. (D) Confocal images of Neuro-2A cells transfected with the sensor vector, the CFP reporter vector and either SCR-132 or miR-132. Scale bars: 70 µm. (PDF) [file pone.0038174.s002.pdf]

Figure S3, Pathania

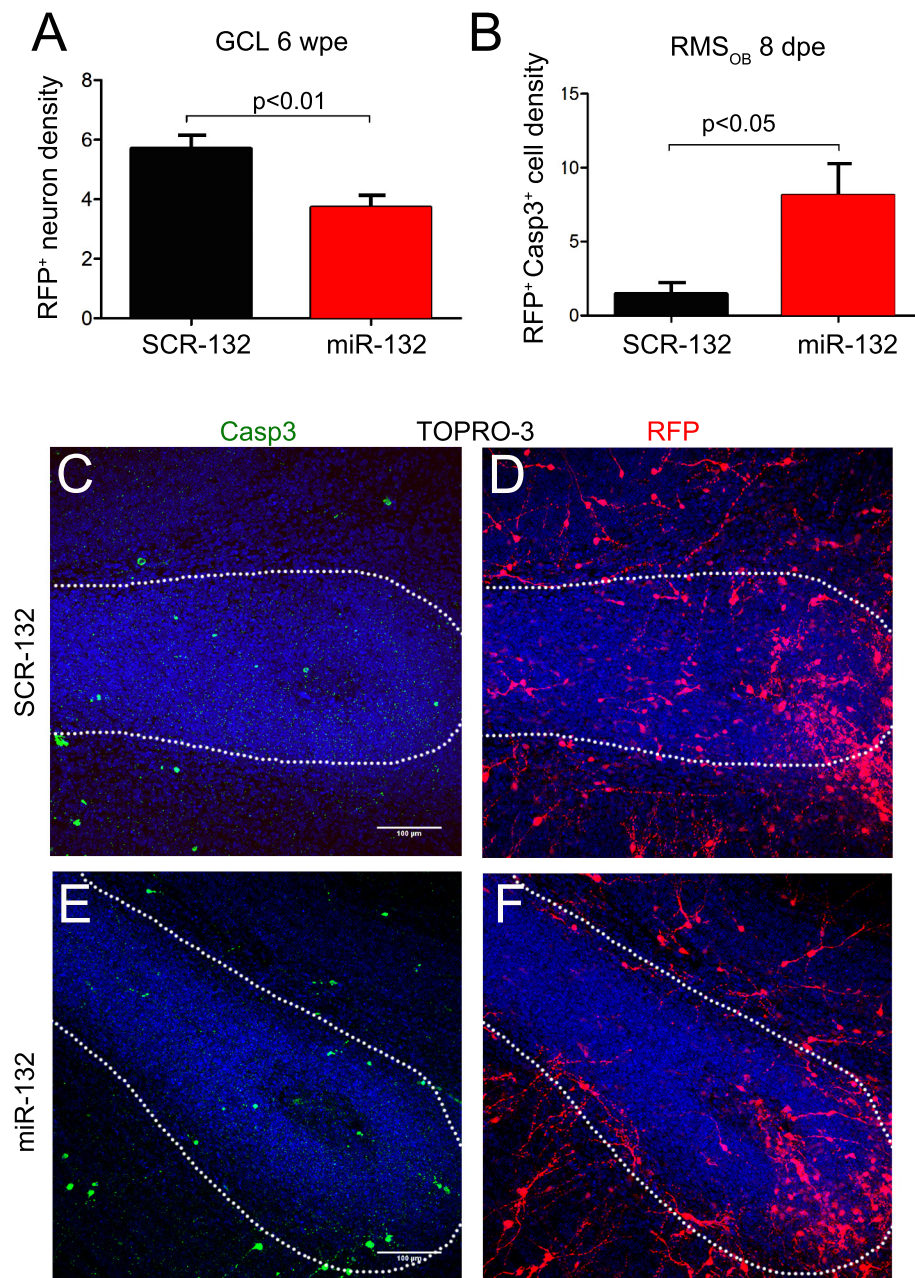

Supplement: Figure S3 — miR-132 overexpression in neuroblasts at birth led to apoptosis. (A) Bar graphs of the RFP+ (i.e. SCR-132, black and miR-132, red) neuron density in the GCL at 6 wpe (N = 3 mice each, 3–4 images analyzed per mouse). (B) Bar graphs of the percentage of RFP+ neuron being activated Caspase 3-positive (Casp3+) in the RMSOB at 8 dpe (N = 3 mice each, respectively). (C–F) Sample images of Casp3 staining (green) with TOPRO-3 (blue, C and E) and RFP staining (red) with TOPRO-3 (blue, D and F) in the RMSOB containing SCR-132 (C and D) and miR-132 (E and F) -expressing newborn neurons. Scale bar: 100 µm. (PDF) [file pone.0038174.s003.pdf]
